# Word difficulty determines the accuracy of regressive saccades in reading

**DOI:** 10.3758/s13423-025-02798-z

**Published:** 2026-01-05

**Authors:** Anne Friede, Albrecht Inhoff, Christian Vorstius, Ralph Radach

**Affiliations:** 1 University of Wuppertal, Wuppertal, Germany; 2 Binghamton University, Binghamton, NY 13902 USA

**Keywords:** Reading, Eye movements, Regressive saccades, Long-range regressions

## Abstract

The current experiment was conducted to study effects of lexical word difficulty on the control of long-range regressive saccades. Participants read single line sentences in German for comprehension and checked for a spelling error that was inserted when the eyes had reached the end of the line. When words were more difficult in terms of orthographic irregularity and lower frequency, this dramatically increased the accuracy of regressions back to these words. If the target was missed, fewer additional saccades and less time were needed until the eyes fixated the target word. The data suggest that more effortful word processing is related to a better representation in visual–spatial memory, enabling more effective programming of regressions.

## Introduction

Natural reading includes eye movements against the direction of reading (which is from right to left in roman and modern Chinese scripts and from left to right in Hebrew and Arabic scripts). These regressive saccades occur with a frequency of between 15 and 25%, depending on materials, reading ability, instructions and other factors (Vitu & McConkie, 2000). There are several possible reasons for the execution of a regression, both on the level of basic visuomotor control and linguistic processing (see Inhoff et al., 2019, for a recent discussion). In the present paper, we focus on long-range regression, targeting words left of the word *n* − 1, which are intended to go back to a specific word on the same line of text. This is a common scenario in normal reading.

Long-range inter-word regressions aid comprehension and therefore ensure reading for understanding (Booth & Weger, 2013; Schotter et al., 2014; Slattery & Rayner, 2009). Words that are difficult to identify are more likely the target of inter-word regressions compared to less difficult words (Kliegl et al., 2004; Vitu & McConkie, 2000). On the lexical level, processing difficulty of a word is usually indicated by the frequency of that word in the language. Words with low frequency are generally fixated longer than words with higher frequency (Henderson & Ferreira, 1990; Inhoff & Rayner, 1986; Kliegl et al., 2006; Rayner et al., 1996; Schuster et al., 2016). The processing time of words is also influenced by frequency or familiarity on the letter level. As an example, manipulating the frequency of initial letter trigrams and quadrigrams within three categories (low, medium, and high orthographic regularity), Radach et al. (2004) found that viewing times increased for less regular word beginnings. Saccade amplitudes are shorter and landing sites shifted to the left for words with irregular beginnings (Hyönä, 1995; Radach et al., 2004; Reichle et al., 2003; Vonk et al., 2002; White & Liversedge, 2004).

Another important reason for inter-word regressions are lexical and/or structural ambiguities within sentences. In this case, inter-word regressions are used for re-inspection and integration of the word meaning into the sentence context (e.g., Bicknell & Levy, 2011; Frazier & Rayner, 1982; Levy et al., 2009; Liversedge et al., 1998; Mitchell et al., 2008). Sentences with structural ambiguity are often referred to as garden path sentences. When it turns out that a prior structural interpretation within a sentence fails, the respective word has to be reassigned to another phrase in the sentence. This type of reanalysis leads to increased viewing times and regression probabilities (see Clifton & Staub, 2011, for a comprehensive discussion). Lexical ambiguity can arise when words have more than one meaning. When the initially assigned meaning (usually the meaning with the higher probability of occurrence) of a word does not match the following context, more regressions are sent back to this word compared to words with only one meaning (Bicknell & Levy, 2011; Carpenter & Danemann, 1981; Frazier & Rayner, 1982).

Just like other processes of reading, the programming of inter-word regressions could be subject to intra-individual differences, anchored in personal characteristics of the reader. Individual differences in text reading were described, for example, by Hyönä et al. (2002) for adult readers and the importance of analyzing individual scanpaths during reading to capture differences in reading performance was emphasized by von der Malsburg et al., (2015; von der Malsburg & Vasishth, 2011). An early account of individual strategies in regression programming has been provided by Frazier and Rayner (1982) who looked at sentences with structural ambiguities: They found that readers either used a *selective reanalysis strategy* and regressed directly to the disambiguation area, or they engaged in a *forward reanalysis strategy*, starting at the beginning of the line to process the whole sentence again. A first analysis of individual differences in regression programming related to reading comprehension was published by Murray and Kennedy (1988) who found differences in their participants (children with an age between 9 and 11 years) according to their reading performance. Good readers usually found the regression target with one single shot regression, regardless of whether the target was near or far away from the starting point of the regression. Poor readers executed significantly less single shot regressions and had to rely on a so-called backward scanning strategy, starting at the end of the sentence and working their way through the sentence with small regressive saccades until the target word was found.

Importantly, the frequency of regressions can be adapted to demands made on the reader. Expecting difficult questions following the reading materials frequently results in more regressions (Weiss et al., 2018; Wotschack & Kliegl, 2013), which are often programmed from the two or three final words of the sentence and led to increased re-reading times (Weiss et al., 2018).

In this paper, we address the question of how the difficulty of a target word may affect the programming of long-range regressions back to this word. To this end, frequency and orthographic regularity of target words were varied within two categories (high vs. low) and a set of sentences was developed so that each sentence included targets that are either difficult or easy to process.

Two opposing hypotheses appear viable. On the one hand, the position of difficult words might be located particularly well during the planning of a regression, because a lot of processing time had been spent on this position within the sentence, creating ample opportunity to store word position information in visuo-spatial working memory. This would lead to the programming of more accurate initial regressions and to a higher rate of single shot regressions that attain the target directly for difficult words as opposed to easy words.

On the other hand, regressing to a more difficult word might be problematic, as less resources for spatial memory encoding could have been available while reading the word with more mental effort. In this case, one would expect that difficult words are targeted by less accurate initial regressions, needing more effort to correct erroneous landing positions and more time to reach the target word. Another possibility is that the difficulty of a word has no effect on the accuracy of regressions. This would suggest that spatial tags might be assigned independently of the linguistic properties of words within sentences.

## Method

### Participants

Fifty-four university students (46 women) with a mean age of 22.1 years (min = 17.8 years, max = 30.8 years) participated in this experiment. All participants were native German speakers and had normal or corrected-to-normal vision. They gave written consent prior the experiment and received course credits for participation. One participant needed to be excluded, because the experiment could not be completed for personal reasons. The study was approved by the ethics committee of the University of Wuppertal (SK/AE 220829) and complied with the ethical standards of the 1964 Declaration of Helsinki regarding the treatment of human participants. The study was not preregistered. The material, data and scripts used for the main model analyses are available online (https://osf.io/4a6ky/).

### Materials

**Fig. 1:**
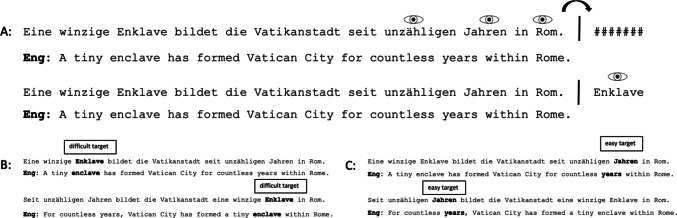
Example sentences out of the experiment. Target words are not marked in any way during the experiment. **Condition A** illustrates the invisible boundary. When crossing this boundary with a saccade, the probe word at the end of the sentence gets visible, which was masked through hashmarks beforehand. **Condition B** represents sentences with a difficult word, each as far (first sentences) and as a near target (second sentence). **Condition C** represents the same sentence frame, but with the easy word being marked as the target word. The first sentence shows the easy target word as a near target, the second sentence as a far target

The materials comprised 100 declarative sentences with a length between 69 and 79 letter spaces and contained 9–14 words. These included a total of 4 practice sentences, 64 experimental sentences and 32 filler sentences. Each sentence contained a target word (experimental sentences) or a filler word (filler sentences). An additional probe word that was identical to the target or filler word in the sentence was positioned five letter spaces to the right of the last word of the sentence. The probe was masked with a sequence of #s (width corresponding to target word length) while the sentence was read, and unmasked when the eyes crossed the center of the area between sentence ending and the string of #s as described in Fig. 1A (the pixel located precisely between the last letter and the first # represented an invisible display change boundary; Rayner, 1975). All targets (and corresponding probes) were nouns with six or seven constituent letters and either part of the subject, object, the adverbial complement or the predicative complement of the sentence, to avoid any training effects for a special grammatical element. Each target word was preceded by an adjective, which was 5–8 characters long, to ensure minimal word skipping of the preceding words and the minimization of spill-over effects.

All 64 experimental sentences were constructed such that each sentence contains one simple and one difficult word as a potential target. In addition, these targets can be in the first third of the sentence (far from the probe at the end of the sentence) or in the last third of the sentence (near to the probe). This is possible in German because parts of sentences can change order easily. We created pairs of sentences so that in one version the target was in the beginning position and in the other version in the near-end position (see Fig. 1B–C) If the target word was located at the beginning, it was preceded by two or three words; if it was near the end, it was followed by one or two words. As an example, each participant would read both the sentences “A tiny enclave has formed Vatican City for countless years within Rome” and “For countless years Vatican City has formed a tiny enclave within Rome” (word-by-word translation). Position and difficulty result in a total of four types of target (easy near, easy far, difficult near, and difficult far). Note that all target nouns were preceded by an adjective and embedded within a noun phrase, regardless of position within sentence or difficulty. The order of the sentences was carefully controlled, so that the two variants of each sentence frame were positioned as far away from each other as possible.

In addition to the 64 experimental sentences, sentence materials also contained 32 filler sentences with a regression target near the center of the sentence (filler word). Filler items met the same criteria as target items. This was used to counteract the development of location specific target expectancies, as the word that matched the probe could have appeared anywhere in a sentence with equal frequency (except for the very beginning and ending).

Word frequency and orthographic regularity were used to define word difficulty. Word frequency is defined as number of word types per million based on the dlexDB word corpus (Heister et al., 2011). High frequency was set to ≥ 150 per million and low frequency to ≥ 1 ≤ 3 words per million. Orthographic regularity is defined for a given word type as the number of types with the same word length and the same initial letter trigram, again based on the dlexDB database (Heister et al., 2011) and was set to > 10 for high and to < 5 for low orthographic regularity. Word length was set to 6–7 letters, to avoid confounding frequency effects with word length (e.g., Kliegl et al., 2004).

Retrieval of high-frequency 6 to 7 letter long nouns (≥ 150 p. M.) in the dlexDB word corpus resulted in a total of 74 hits. These candidate words were sorted in descending order of orthographic regularity and the 52 words that had an orthographic regularity of > 10 were selected. For the difficult words, a normalized frequency of ≥ 1 ≤ 3 and an orthographic regularity of < 5 were specified. For 6–7 letter long nouns, this resulted in an item pool of 128 words. These two lists were used to construct the sentence materials for the experiment. Altogether, easy words had a mean frequency of 307.5 (*SD* = 225.3) and a mean orthographic regularity of 50 (*SD* = 22.8) per million, whereas difficult words had a mean frequency of 1.97 (*SD* = 0.7) and a mean orthographic regularity of 2.5 (*SD* = 1.5) per million.

### Procedure

Participants were asked to read the single line sentences silently, at a comfortable pace and for good comprehension, so that they could answer sentence comprehension questions that would occur after the reading of a subset of sentences. Participants were also instructed to look at the masked probe location to the right of the sentence, after they had read the sentence. They were told that this would reveal a probe word corresponding to a target word in the previously read sentence. To entice readers to execute a regression to the target and to make this task meaningful, they were asked to determine whether the target was correctly or incorrectly spelled when re-read. Half of the targets were unchanged and correctly spelled during re-reading, the other half were misspelled by changing a single non-initial letter of the word (inserted after the sentence had been read and the invisible boundary at the end of the sentence had been crossed). In the construction of spelling errors vowel replaced vowel and consonant replaced consonant. Descending, ascending and baseline letter were exchanged by the same category of letters, thus maintaining the original length and word form (e.g., “Arbeit” became “Arteit”). These minimal changes were always implemented at near-center locations within words, ensuring that the resulting pronounceable non-words constituted visually non-conspicuous targets for regression saccades. Error detection was very good, with an average of 97% (min = 94%, max = 100%) correct key presses. Out of the 100 sentences the first four served as practice items to make sure that all instructions were completely understood. Sentences were presented in Courier New with a font size of 15 pt in black on a light grey background on a 21-inch flat-panel monitor with a resolution of 1,680 × 1,050 pixels and a refresh rate of 120 Hz. Viewing distance was set to 68 cm, so that every letter occupied a width of 0.3° of visual angle. Eye movements were recorded using an SR EyeLink® 1000 plus video-based eye tracking system (SR Research, Toronto, Canada) and a sampling rate of 2000 Hz. A chin rest and forehead rest minimized head movements. At the beginning and at several points during the experiment a 3-point calibration, followed by a validation routine, was performed. A drift check before the start of each sentence ensured accuracy between calibrations.

To ensure that participants read for understanding, comprehension questions were asked after approximately every sixth sentence, which had to be answered orally. The questions were designed to address either simple (location, actor, object, attribute) or complex (time, cause) semantic relations, requiring careful reading for understanding (Radach et al., 2008). Answers were noted by the experimenter. The average proportion of correct answers was 18.96 for a total of 24 questions (min = 14, max = 24). The session lasted about 30–45 min, depending on the reading speed of the individual participant.

How can regressions during sentence reading be studied? One way to trigger many regressions is to use garden path sentences (e.g., Bicknell & Levy, 2011; Ferreira & Henderson, 1991; Frazier & Rayner, 1982; Meseguer et al., 2002; Sturt & Kwon, 2018). However, the analysis of regressions in implausible sentences revealed that most regressions did not originate from the mismatching verb, but from the end of the sentence (Sturt & Kwon, 2018, used implausible sentences like the following: “There was an old house that John had ridden when he was a boy”; the verb “ridden” marks the point at which the sentence becomes implausible. One would expect most regressions launched from that critical word, while in their experiment most of the regressions were programmed from the end of the sentence.). In addition, saccades did often not selectively return to the phrase causing the confusion (Mitchell et al., 2008), indicating that in the case of garden path sentences the goal of regressions is generally not under the experimenters’ control.

We decided to examine the metrics of regressive saccades using a dual task situation. The primary task was reading for meaning in preparation for comprehension questions. As a secondary task, participants checked for spelling errors in words that were presented after the eye crossed an invisible boundary to the right of the line of text. During reading all word were correct, and spelling error were created in 50% of the cases during the display change (see Fig. 1). This task was inspired by the seminal work of Kennedy and Murray (1987) and Kennedy et al. (2003). They asked readers whether a certain word had been in a line of text just read, but eliciting regressions only in 10 to 24% of all cases.

Can our task reflect regressions occurring in natural reading? Results obtained by Inhoff and Weger (2005) indicate that the size of regressions to near and far targets within one-line sentences is identical, no matter if regressions occurred naturally or if participants were instructed to regress to a certain target. Furthermore, the request to regress from a position close to the end of a sentence is similar to naturally occurring regressions in particular when difficult text has been read. Weiss et al. (2018) showed that regressions were often initiated near the final words of the sentence (see also Inhoff et al., 2005). In an experiment by Tiffin-Richards and Schroeder (2018), the authors found, that the proportion of regressions initiated at sentence boundaries was greater in adults than in children, “suggesting that the use of sentence endings to initiate regression increases with reading experience and proficiency” (p. 1059).

### Data selection

Saccades and fixations (recorded during the reading task) were classified online using the EyeLink software. Raw data output from the recording system was further processed using the software suite EyeMap (Tang et al., 2012) that generated a wide range of oculomotor indices for each word of the sentence. Since the study sought to examine regression guidance, four regression-related oculomotor measures were extracted and analyzed: (1) initial regression size, consisting of the size of the first saccade (in letter spaces, LS) from the probe toward the target location; (2) the regression error, comprising the distance (in LS) between the initial landing position and the center of the target; (3) the number of regressions (first regression and all following saccades) that were needed to reach the target word after the probe word was fixated; and (4) total regression time, comprising the interval (in ms) between the onset of the first regression toward the target and the fixation of the target.

Values of all parameters had to be log-transformed for statistical analyses to better fit normal distributions. Figures and tables report non-transformed values for ease of interpretation. To remove outliers, the following exclusion criteria were used: for length of initial regression outliers with log values of < 2.0 (> 80 letter spaces; 0.6%), for regression errors outliers with log values of ≥ 2.0 (< 0.1 letter spaces; 0.6%), for number of regressions outliers with log values of > 2.2 (> 10 fixations after crossing the boundary back into the sentence; 1.3%), and for total regression time outliers with log values of < 4.5 and > 7.7 (< 07 ms and > 2,192 ms; 0.8%) were excluded.

### Data analysis

In a first step, the four regression measures from the reading task were analyzed separately to identify significant predictors of readers’ regressions. The second step comprised a qualitative selection of regression strategies (types) and an examination of regression accuracy as a function of regression strategies. Linear mixed models (LMM), as implemented in the “lmer” function from the *lme4* package (Bates et al., 2015) in the R environment for statistical computing (Version 2022.12.0 + 353; R Core Team 2022), were used to analyze the four regressions measures. The LMMs included the fixed effects difficulty (difficult vs. easy) and position (near vs. far) and interaction of these factors. The LMM started with a maximal random effect structure but this structure was simplified as maximal models failed to converge. Specifically, maximum likelihood comparison revealed that only the variance components of participant and item intercepts and participants’ slope for the position effect improved the model. The fixed effects of position and difficulty and their interaction improved the prediction of the statistical model and were consequently not removed.

## Regression strategies

**Fig. 2:**
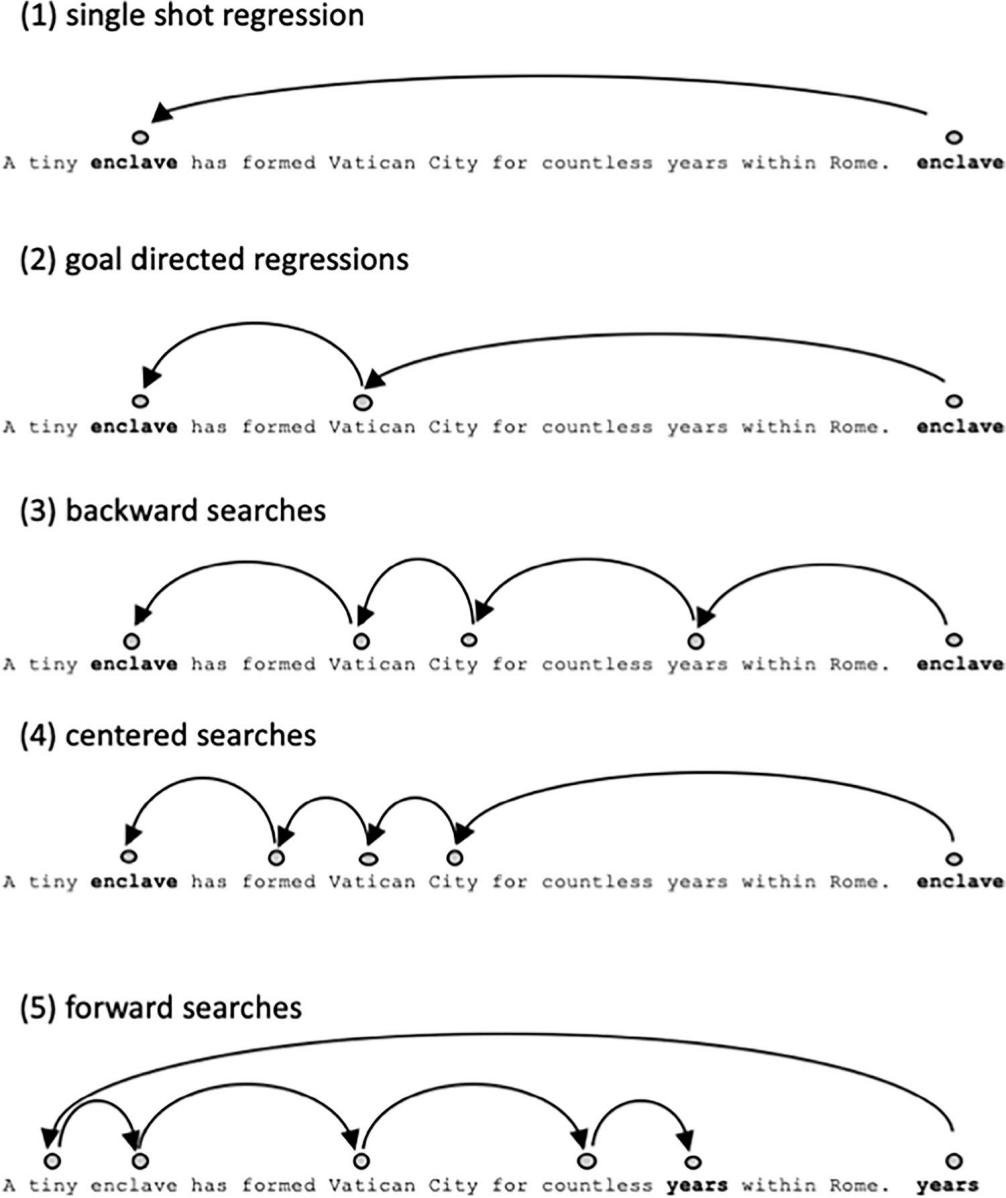
Representation of all categories of scanpaths from the probe word to the target word. Single Shot Regressions **(1)** immediately attain the target, goal-directed regressions **(2)** describe regressions with a slight over- or undershoot followed by only one corrective saccade, all other scanpaths describe search strategies depending on the landing position of the first regression back into the sentence, which can be either at the end of the sentence **(3)**, in the middle of the sentence **(4),** or at the sentence beginning **(5)**

Visual inspection of eye movement patterns during every trial allowed the classification of five different regression strategies: (1) single shot regressions include every regression that immediately attains the target (22.2%); (2) goal directed regressions are regressions with a slight under- (at least 50% of the distance) or overshoot (at most 150% of the distance) followed by only one corrective saccade (18.2%); (3) backward searches constitute a searching process starting in the last third of the sentences and needing at least two corrective saccades to attain the target word. (18%); (4) centered searches describe a pattern where the first regression is targeted towards the center of the sentences (middle third), followed by at least two corrective saccades (29.6%); (5) forward (beginning-to-end) searches start in the first third of the sentences and also include at least two corrective saccades (5.1%). In 0.6% of all cases, targets were never reached by a regression, hence button presses were executed before attaining the target. Finally, in 6.4% of the trials regressive searching behavior could not be captured within the classification and remained unclassified (see Fig. 2).

**Fig. 3:**
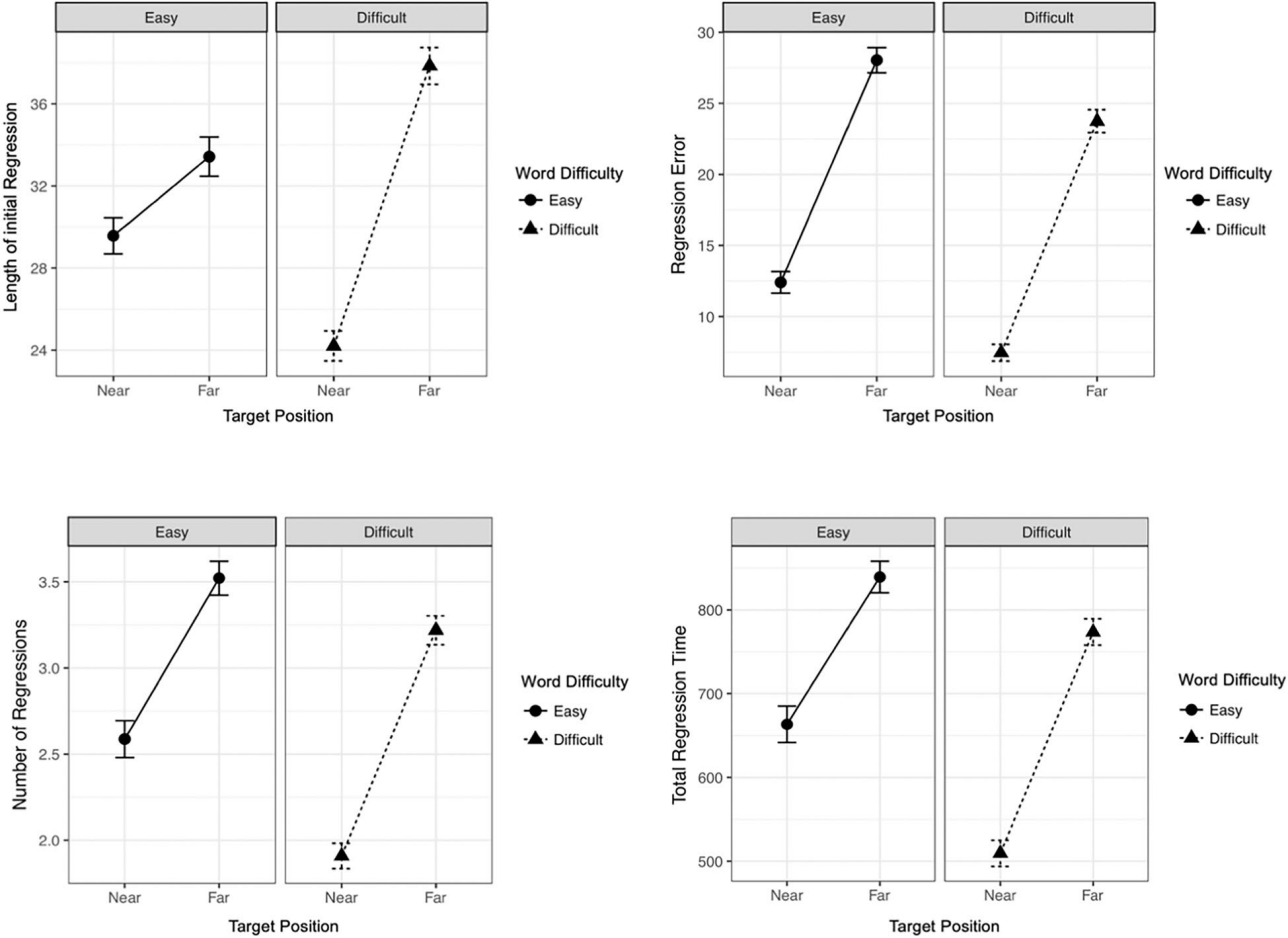
Means of length of initial regressions, regression error, number of regressions and total regression time as a function of word difficulty and target position

We would like to note that the term “strategy” does not imply any active conscious decision by reader. It is common in eye movement research on reading to use the term for automated visual processing and visuomotor programming routines (e.g., O’Regan, 1990). Our “single shot” and “goal directed” strategies are functionally similar, with the main difference being that in one case accuracy is perfect and in the other case a near-perfect primary saccade is followed by a corrective secondary saccade. We decided to maintain the definition of the single shot strategy as introduced in the seminal work by Murray and Kennedy (1988) to enable comparison of results. An alternative is to consider our strategies (1) and (2) jointly as an “accurate regression strategy,” but this would come at the cost of losing information in our analyses (see Fig. 3 for a breakdown of how frequently the strategies occur).

## Eye-movement measures

To determine the processing time of difficult and easy target words, temporal eye movements representing early, intermediate and late linguistic processing were used (see Radach & Kennedy, 2004; Rayner, 1998, for overviews). First fixation duration and single fixation duration are reported as early measures. These two first-pass measures are generally assumed to reflect orthographic and early lexical processing (Hyönä et al., 2003; Inhoff & Radach, 1998). The sum of all fixations on a word, until this word is left for the first time, is reported as gaze duration and, as an intermediate measure, reflects later stages of word processing such as lexical access.

Additionally, the later-pass measures re-reading time and total viewing time are reported. The re-reading time describes the duration of all fixations made on the word after leaving it for the first time and total viewing duration is the sum of gaze duration and re-reading time and reflects the duration of all fixations made on a word. These later measures are associated with comprehension issues on sentence and passage level and the integration of the meaning into previously read materials (Radach & Kennedy, 2013).

In first pass reading, 304 (9.6 percent) of all target words were skipped and never fixated. Of these, 78 words were classified as difficult and 226 were classified as easy words. Again, all temporal eye movement measures were log-transformed for a better fit of normal distribution. Exclusion criteria were log-values of > 4.3 and < 6.5 (> 665 ms, < 74 ms; 1.2%) for single fixation duration, > 4.0 and < 6.2 (> 493 ms, < 55 ms; 1%) for first fixation duration, > 4.3 and < 6.7 (> 812 ms; < 74 ms; 1.3%) for gaze duration, < 5.5 (< 245 ms; 0.5%) for total viewing duration and > 5.0 (< 148 ms; 1.4%t) for re-reading time.

## Results

### Effects of word difficulty

The manipulation of word difficulty leads to distinctive differences in word processing, as demonstrated by temporal eye movement measures. Easy and difficult words differed significantly in single- and first fixation duration, gaze duration, re-reading time, and total viewing time (see Table 1).

**Table 1:** Means and standard error for basic temporal eye-movement parameters for easy and difficult words (in ms) and results of the linear mixed model analysis (LMM)

	*M* (*SE*)		Estimate	*SE*	/*t*/-value
	Easy	Difficult			
Single fixation duration	216 (1.9)	249 (2.6)	0.138	0.018	7.78***
First fixation duration	185 (5.3)	212 (4.3)	0.123	0.034	3.608***
Gaze duration	232 (2.4)	289 (3.3)	0.2	0.023	8.596***
Total viewing duration	804 (6.7)	921 (8.5)	0.126	0.02	6.448***
Re-reading time	575 (6)	632 (7)	0.09	0.02	4.474***

***p <.001, *M* = Mean, *SE* = Standard Error, Easy = easy words, Difficult = difficult words

Evidently, difficult words need to be processed with substantially more effort during all stages of word processing and are more likely the target of regressions (hence the longer re-reading time). These results confirm that the distinction between easy and difficult words is effective, allowing for subsequent analyses of regression accuracy on the basis of word difficulty.

### Effects of word difficulty on regression measures

Table 2 provides means and standard deviations for all dependent variables. The initial regression was significantly longer for far (*M* = 35.5, *SE* = 0.3) than for near (*M* = 26.9, *SE* = 0.3) targets, confirming spatial selectivity for target position (*b* = 0.29, *SE* = 0.029, *t* = 10.056, *p* <.001). An interaction effect between position and difficulty revealed that spatial selectivity is especially pronounced for difficult words (*b* = 0.34, *SE* = 0.0483, *t* = 7.043, *p* <.001), leading to a significant larger difference in regression length between far and near targets for difficult (14 LS) than for easy target words (3.6 LS; see Fig. 3). This results in a smaller regression error for difficult than for easy word (*b* =  − 0.44, *SE* = 0.054, *t* =  − 8.185, *p* <.001), which is especially pronounced for near difficult words (*b* = 0.45, *SE* = 0.1129, *t* = 3.961, *p* <.001).

**Table 2:** Means and standard error for amplitude of initial regressions (in letter spaces), regression error (in letter spaces), total number of regressions and total regression time (in ms) as a function of word difficulty (easy vs. difficult) and target position (near vs. far)

		Near		Far	
		Easy	Difficult	Easy	Difficult
Length of initial Regression	*M*	29.6	24	33.2	38
	*SE*	0.4	0.4	0.5	0.5
Regression Error	*M*	12.5	7.4	28.2	23.4
	*SE*	0.4	0.3	0.4	0.5
Number of Regressions	*M*	2.6	1.9	3.5	3.2
	*SE*	0.06	0.05	0.05	0.05
Total Regression Time	*M*	660	508	835	774
	*SE*	12	10	10	10

*M* Mean, *SE* Standard Error

Mean distance from the probe word to the end of near targets was 14.9 letter spaces, and 57.4 letter spaces to the end of far targets

Correction processes in cases when initial regressions did not land on the target were generally influenced by word difficulty (see Fig. 3). Difficult words resulted in fewer regressions (*b* =  − 0.199, *SE* = 0.025, *t* =  − 7.921, *p* <.001) and less total regression time (*b* =  − 0.175, *SE* = 0.022, *t* =  − 7.926, *p* <.001) to eventually attain the target word.

Both variables interact with target position. For both variables, the differences are significantly larger for near targets (number of regressions: 0.7, *b* = 0.197, *SE* = 0.054, *t* = 3.631, *p* <.001, total regression time: 152 ms, *b* = 0.182, *SE* = 0.046,* t* = 3.965, *p* <.001) than for far targets (number of regressions: 0.3, total regression time: 61 ms).

Target position had a main effect, too. Near targets generally required less regressions (*M* = 2.2, *SE* = 0.04,* b* = 0.5, *SE* = 0.047, *t* = 10.744, *p* <.001) and less total time (*M* = 586 ms, *SE* = 8.1, *b* = 0.396, *SE* = 0.038, *t* = 10.387, *p* <.001) than far targets (number of regressions: *M* = 3.4, *SE* = 0.03, total regression time: *M* = 806.8 ms, *SE* = 7.1) to be reached. See Table 3 for summarized Results of all LMM analyses.

**Table 3:** Results of the LMM analyses

	Factor	Estimate	Standard Error (*SE*)	/*t*/-Value
Length of initial Regression	Target Position	0.28971	0.02881	10.056***
	Difficulty	− 0.02487	0.02238	− 1.112
	Target Position X Difficulty	0.33996	0.04827	7.043***
Regression Error	Target Position	1.37226	0.11062	12.405***
	Difficulty	− 0.44422	0.05427	− 8.185***
	Target Position X Difficulty	0.44729	0.11291	3.961***
Number of Regressions	Target Position	0.50061	0.04659	10.744***
	Difficulty	− 0.19905	0.02513	− 7.921***
	Target Position X Difficulty	0.19696	0.05424	3.631***
Total Regression Time	Target Position	0.39555	0.03808	10.387***
	Difficulty	− 0.17516	0.02210	− 7.926***
	Target Position X Difficulty	0.18281	0.04611	3.965***

***p <.001

### Regression strategies

To test the influence of word difficulty on regression strategies, a repeated-measures ANOVA with the within-subject factors regression strategy (single shot, accurate regression, centered search, backward scanning and forward scanning) and word difficulty (easy vs. difficult) was carried out. Degrees of freedom were corrected using Greenhouse–Geiser estimates of sphericity, χ^2^(20) = 226.928, *p* ≤.001, ε =.487. Word difficulty had a significant influence on the used strategies, *F*(4.223, 219.581) = 23.637, *p* <.001. Post hoc tests with Bonferroni correction revealed that difficult words were targeted by a higher number of single shot regressions (*M* = 8.38, *SD* = 3.8) than easy words (*M* = 4.94, *SD* = 3.3), *p* ≤.001, *d* =.818. The amount of goal directed regressions also increased, but did not reach significance (*p* =.109). Easy words were more likely reached by search strategies, represented by a significantly higher amount of centered search (easy: *M* = 10.11, *SD* = 5, difficult: *M* = 7.6, *SD* = 4.1), *p* ≤.001, *d* =.487, backward search (easy: *M* = 6.68, *SD* = 4.3, difficult: *M* = 4.1, *SD* = 2.8),* p* ≤.001, *d* =.752 and forward search (easy: *M* = 1.87, *SD* = 2.1, difficult: *M* = 1.2, *SD* = 1.2), *t*(52), *p* ≤.05, *d* =.324 regression strategies in easy target words.

**Fig. 4:**
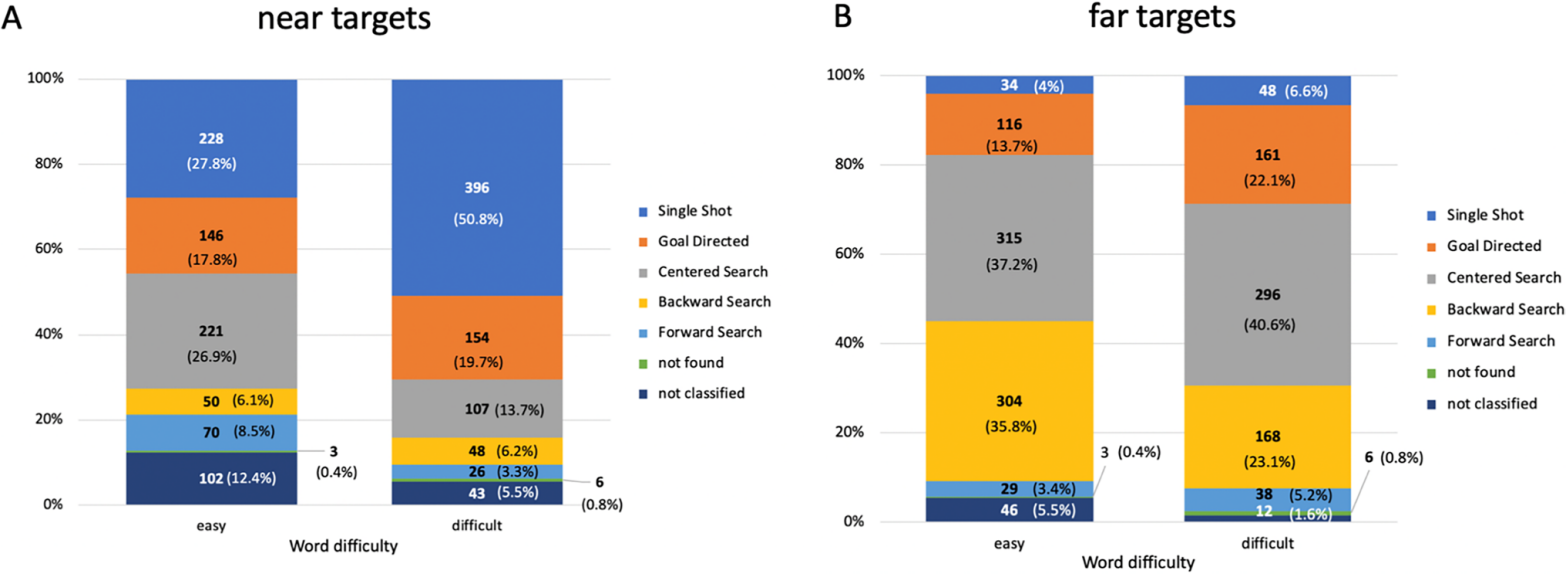
Distribution of regression strategies (both number of cases and percentage) as a function of word difficulty for **A**: near targets and **B**: far targets. For near targets (A), data for easy words include 820 executed regressions toward targets, while results for difficult words are based on 753 regressions aimed at target words. For far targets (**B**), data for easy words include 847 regressions toward targets, while results for difficult words are based on 729 regressions aimed at target words. (Color figure online)

Additional repeated-measures ANOVAs tested for differences in the use of strategies for near and far targets. For near targets, word difficulty influenced the preferred use of regressions, *F*(3.866, 201.030) = 26.818, *p* ≤.001 (see Fig. 4). Degrees of freedom were again corrected using Greenhouse–Geiser estimates of sphericity, χ^2^(20) = 285.519, *p* ≤.001, ε =.354. Subsequent post hoc tests with Bonferroni correction indicated that for near targets the number of single shot regressions dramatically increased with difficult targets, *p* ≤.001, *d* =.85 (easy: *M* = 4.3, *SD* = 3.2, difficult: *M* = 7.47, *SD* = 3.5). The resulting lower proportion of search strategies is reflected in a significantly reduced use of centered search regressions toward difficult (*M* = 2.02, *SD* = 2.1) compared to easy targets (*M* = 4.2, *SD* = 3.2), *p* ≤.001, *d* =.762, whereas the proportion of backward searches remained very similar, *p* =.814.

With far targets, the ANOVA again revealed a significant influence of word difficulty on strategy, *F*(3.218, 167.315) = 14.651, *p* ≤.001, also with corrected degrees of freedom, χ^2^(20) = 327.054, *p* ≤.001, ε =.389 (see Fig. 4). A higher proportion of accurate regressions was reflected in a significant higher number of goal directed regressions in difficult (*M* = 3.0, *SD* = 2.4) than in easy targets (*M* = 2.2, *SD* = 2.2), *p* ≤.05, *d* =.340, while the number of single shot regressions remained constant, *p* =.075. Furthermore, the number of backward searches increased with easy targets (*M* = 5.7, *SD* = 3.9, difficult targets:* M* = 3.2, *SD* = 2.3), *p* ≤.001, *d* =.793, but the number of centered searches was not different between easy and difficult targets, *p* =.447. Again, Bonferroni correction was included in post-hoc tests.

### Discussion

In this experiment, we provide a comprehensive metrical description of regressive saccades after the reading of sentences. We confirm the seminal findings by Kennedy and Murray (1987) and Kennedy et al. (2003) that long-range regressions can be remarkably accurate and provide a description of strategies used by readers to attain the saccade target. The accuracy of long-range regressions has been expressed in the spatial coding hypothesis by Kennedy and Murray. The assumption being, that during reading words will be linked to a spatial index of their position that can be retrieved and used for regression targeting. A clever way to implement this idea computationally can be seen in the OB1 model, where activated words are mapped onto a spatiotopic sentence-level representation (Snell et al., 2018).

The main goal of the present work was to go a step further and examine the influence of word processing difficulty on the planning and execution of regressions. The main finding was that difficult words dramatically increased the precision of long-range regressions and were targeted more often by accurate regressions. As a result, less pronounced search processes took place, reflected in fewer regressions and less regression time. This is particularly evident in the proportion of regression strategies, with almost twice as many single shot regressions in the near condition and substantially more goal directed saccades in the far condition for difficult words.

The results of this experiment also confirmed an effect of target distance: initial regressions are longer for far than for near targets, indicating spatial selectivity of long-range regressions and the retrieval of word location information from spatial memory. At the same time, far targets are in general more error-prone, as indicated by a higher number of regressions and a longer total regression time. These results are in line with earlier findings from Inhoff and Weger (2005), Weger and Inhoff (2007) and Guérard and colleagues (2012).

This effect of spatial selectivity of initial regressions is much more pronounced for difficult targets. Apparently, the ability to adapt the length of the initial regression to the distance of the target word increases with the processing time spent on a word. Far and difficult targets lead to longer regressions than far and easy targets, whereas near and difficult targets evoke shorter regressions than near and easy targets, resulting in more regressions attaining the word without the need for landing position correction. These findings are the first to indicate directly that the mental effort invested in word processing is closely related to the way how spatial word location information is stored in memory and utilized for the programming of regression saccades.

This assumption goes hand in hand with classical findings about the remembering of easy and difficult materials. Prior work on the desirable difficulty effect suggests that difficult learning conditions, which engage the learner more deeply and slow learning speed, can have a positive effect on memory retention and transfer after training (Bjork, 1994; Bjork & Bjork, 2014). This effect can be transferred to reading. When reading, gaps in text coherence, which are making reading more difficult and challenge readers to consider inferences and draw conclusions, are advantageous for learning, at least for readers with adequate background knowledge (Kintsch, 1994). For competent readers, texts with insufficient coherence can activate prior knowledge, helping to anchor new information into memory (Fulmer et al., 2015; McNamara, 2001; McNamara et al., 1996). In the context of the present experiment, it could also be the case that location information for difficult words is more easily retrieved due to deeper processing during the inflated prior processing time.

Rather than being a product of word-level processing, the regression advantage for more difficult words might also originate on the level of sentence processing. It may be the case that difficult words are preferentially stored in spatial memory because they are more likely to cause comprehension difficulties. Consequently, regressions to such locations need to be programmed more frequently to clarify any comprehension problems. In contrast, easy words leave less explicit traces in spatial memory, because they are not likely associated with comprehension issues and necessary repairs. In harmony with this view, prior work has shown that sentences with lexical and syntactical ambiguities (Frazier & Rayner, 1982; Mitchell et al., 2008; Rayner & Duffy, 1986) provoke more regressions from the disambiguating sentence region back to an ambiguous target word.

A final piece of evidence supporting this view comes from work on the “inhibition of return” (IOR) phenomenon, where the return of attention to a target that has been attended before leads to an increased latency compared to a target that has not been attended previously (Posner & Cohen, 1984). This effect has also been applied to reading, based on longer fixation durations prior to regressions back to previously fixated words in comparison to previously skipped words (Rayner et al., 2003). As recently shown by Eskenazi and Folk (2017), this IOR-like effect is virtually absent for regressive saccades caused by comprehension difficulties in the case of ambiguous words. This result points to the possibility that readers could anticipate that difficult words may lead to uncertainties in understanding and that retaining information on their location may benefit the resolution of later comprehension difficulties.

As pointed out above, the main conclusion we draw from our work is that the reading of difficult words is related to establishing a more effective tag in visuo-spatial memory that can help moving the eyes back when needed. At this point we cannot be certain whether the more effortful linguistic processing of difficult words directly caused the targeting advantage for these words or, alternatively, the encoding of word locations as potential future regression targets takes advantage of the extra time available while viewing the respective word. We believe that first alternative to be more likely, but the final decision will need to come from more detailed experimental work on this very interesting issue.

Our work is the first to provide a comprehensive description of regression strategies that readers may utilize during natural reading. However, in our experiment, a specific target word is always specified as the goal for reinspection. Even though Vitu and McConkie (2000) concluded from the analysis of a large corpus of text reading data that most regressive interword saccades are indeed aimed at specific target words, this will not be true for all observations. Our own strategy analyses indicate that even when a target word is specified, this information is sometimes lost or not used, which is presumably the reason for centered, backward and forward searches (see Figs. 2 and 3). These strategies will likely occur more often in natural reading situations when the identity of the target word or region is initially opaque. Therefore, our design should be taken as an ideal model, approximating the limits of performance for long distance regressions during reading.

## Data Availability

The material, data and scripts used for the main model analyses are available from https://osf.io/4a6ky/
